# Hot-Air Thermal Pre-Treatment as a Rejuvenation Strategy for Long-Stored Low-Amylose Aromatic Rice: Effects on Drying Kinetics, Microstructure, and Cooked-Rice Texture

**DOI:** 10.3390/foods15193376

**Published:** 2026-09-23

**Authors:** Pisut Maichoon, Prasan Choomjaihan

**Affiliations:** Department of Biosystems and Agricultural Engineering, School of Engineering, King Mongkut’s Institute of Technology Ladkrabang, Bangkok 10520, Thailand; pisut.maichoon@gmail.com

**Keywords:** aged rice, rice aging, thermal pre-treatment, water absorption kinetics, effective water diffusivity, cooked-rice texture, multi-objective optimization, entropy weight method

## Abstract

This study examined whether hot-air thermal pre-treatment can rejuvenate 12-month-aged Khao Dawk Mali 105 (KDML 105) rice by inducing surface cracks that promote water uptake. Aged rice was dried at temperatures of 40, 60, 80, 100 and 120 °C to target moisture contents of 10%, 8%, 6% and 4% wet basis (w.b.), giving 16 combinations, with drying times predicted from thin-layer drying models. The drying kinetics, crack morphology, water absorption, color and cooked-rice texture were compared with those of fresh rice (stored for 2 months) and untreated aged rice. The Two-term and Page models described drying accurately (R^2^ > 0.982, RMSE < 0.039). Light-transmission imaging revealed a surface crack network in all pre-treated samples. Water absorption followed the Peleg model, with the initial absorption rate (1/K_1_) rising from 0.026%/s in untreated aged rice to 0.099–0.198%/s, and the effective water diffusivity (D_eff_) from 0.63 to 1.04–1.35 × 10^−10^ m^2^/s. Drying to 10% w.b. reduced the hardness from 25.09 to 16.28–18.53 N, comparable to fresh rice (16.70 N), whereas 4% w.b. increased it again (23.27–25.11 N); stickiness was not restored. Multi-objective optimization identified 10% w.b. as the determining factor at every drying temperature, with T40–M10 scoring the highest (CI = 0.509; relative CI = +0.118). Terminating drying at a target moisture content of 10% w.b., rather than the drying temperature applied, was the primary determinant of successful textural rejuvenation, whereas stickiness remained unrestored under every condition tested.

## 1. Introduction

Rice is a primary source of carbohydrates and energy for the global population, and starch constitutes approximately 80–90% of its dry weight [1]. Consumption preferences vary widely and are governed by the texture of the cooked product [2,3], with hardness and stickiness being the two attributes that most strongly determine consumer acceptance [4]. The majority of Asian consumers generally prefer rice that is soft and sticky, that is, low in hardness and high in stickiness—a combination particularly pronounced in Khao Dawk Mali 105 (KDML 105), a cultivar known for both its soft cooked texture and its characteristic aroma [5]. After prolonged storage, however, milled rice undergoes a natural aging process that alters the kernel chemically and physically and produces a firmer cooked texture, with hardness increasing and stickiness decreasing markedly [6,7,8,9]. This deterioration directly affects cooking quality and reduces consumer satisfaction and acceptance [10,11].

Aging occurs in all rice varieties, but the severity and pattern of the accompanying physicochemical changes depend on the physical and biochemical characteristics of each variety [12,13]. In general, milled rice stored for longer than three months begins to show clear deterioration in its chemical and physical properties and is referred to as aged rice [12]. In KDML 105, aging leads to a loss of the characteristic aroma, changes in color and a decrease in viscosity; these changes originate in the reorganization of starch, protein and lipid structures at the molecular level within the kernel [14,15].

Among these changes, the loss of texture is the main obstacle to eating quality. The increase in hardness after cooking has been attributed to hardening of the protein network together with changes in the fine structure of starch, which accelerate retrogradation as the cooked rice cools [16,17]. The decline in stickiness has been attributed to reduced water solubility of the starch: lipids within the kernel degrade to free fatty acids, which associate with amylose to form amylose–lipid complexes with a V-type crystalline structure that restrict water absorption and swelling of the starch granules [13,18].

Because these changes are predominantly structural, physical treatments that alter the kernel structure represent a plausible means of reversing them. Reducing agents restore the hardness and stickiness of cooked aged rice to levels comparable with fresh rice by cleaving protein disulfide bonds and promoting the separation and swelling of starch granules, which confirms that restricted granule swelling and separation underlie the textural deterioration of aged rice [6]. Ultrasonic treatment achieves a similar disaggregation of tightly bound starch granules by cavitation, although this has been demonstrated only in rice flour rather than in intact kernels [19]. Among physical methods, thermal treatment is the simplest to implement at scale [20]. Hot-air and infrared heating have been shown to reduce the hardness of cooked aged rice through heat-induced surface fissures that accelerate water absorption into the kernel core, whereas microwave heating is less effective [21]. However, stickiness was not improved by any of these thermal methods [21], and it has likewise been identified as the attribute most resistant to restoration by chemical means [6].

The heating conditions in previous work were defined by exposure time at a single high temperature, so the thermal load was not regulated with reference to the state of the kernel. Terminating heating at a defined moisture content, rather than after a fixed interval, would allow the thermal load to be controlled directly and would permit lower drying temperatures to be evaluated. The combination of drying temperature and endpoint moisture content that induces sufficient cracking to accelerate water absorption without compromising other quality attributes has not been established. This study therefore evaluated the effects of drying temperature and target moisture content during thermal pre-treatment on surface crack formation, water absorption, cooked-rice texture and color in aged KDML 105. The conditions were then compared within a multi-objective optimization framework to identify those bringing the properties of aged rice closest to those of fresh rice.

The specific objectives of this study were therefore as follows: (i) to establish thin-layer drying models for aged KDML 105 rice at 40–120 °C and to use them to prepare samples at defined target moisture contents; (ii) to characterize the resulting surface cracking, water-absorption kinetics, cooked-rice texture and color; and (iii) to identify, through entropy-weighted multi-objective optimization, the combination of drying temperature and target moisture content that brings the properties of aged rice closest to those of fresh rice.

## 2. Materials and Methods

### 2.1. Raw Material Preparation and Initial Characterization

Commercial milled KDML 105 rice samples represented low-amylose aromatic rice and were obtained from a local merchant (Bangkok, Thailand). The rice samples were divided into two groups according to storage duration:

- Fresh rice (control): milled rice stored for 2 months after harvest;
- Aged rice: milled rice stored for 12 months after harvest.

Both groups were kept at room temperature (27 ± 2 °C) and 50–60% relative humidity in plastic containers sealed with rubber gaskets to prevent moisture exchange with the surrounding air. Moisture content was determined by air-oven drying at 105 °C to constant weight, a procedure adapted from AOAC Official Method 945.15 (Loss on Drying (Moisture) in Cereal Adjuncts) and applied in previous studies of stored and processed rice [22,23,24]. The initial moisture content of the aged rice was 12.33 ± 0.18% wet basis (w.b.).

### 2.2. Drying Kinetics, Mathematical Modeling, and Sample Preparation

The experiment consisted of two stages: determination of the drying kinetics to establish a drying-time prediction model, and application of that model to prepare samples at the target moisture contents.

#### 2.2.1. Thin-Layer Drying Experiment and Mathematical Modeling

Aged rice samples of 300 g were dried in a hot-air oven (UF260, Memmert, Schwabach, Germany) at 40, 60, 80, 100 and 120 °C. This range was selected to span conditions from low-temperature drying, representative of ambient or solar drying practices, to the higher temperatures typical of industrial hot-air dryers, and to bracket the reported gelatinization onset temperature of KDML 105 (approximately 70–78 °C) so that both sub-gelatinization and supra-gelatinization thermal regimes were represented among the tested conditions. Sample weight was recorded at intervals throughout drying, and the moisture data were converted to the moisture ratio (MR) using Equation (1):

MR = (M_t_ − M_e_)/(M_0_ − M_e_), (1)

where M_t_ is the moisture content at time t (%d.b.), M_0_ is the initial moisture content (%d.b.), and M_e_ is the dynamic equilibrium moisture content (%d.b.).

The MR data were fitted by non-linear least-squares regression to five thin-layer drying models:

**Newton:** MR = exp (−kt),          (2)

**Page:** MR = exp (−kt^n^), (3)

**Henderson and Pabis:** MR = a · exp (−kt),                          (4)

**Logarithmic:** MR = a · exp (−kt) + c,    (5)

                     **Two-term:** MR = a · exp(−k_0_t) + b · exp(−k_1_t), (6)

where n in the Page model is a dimensionless empirical constant that accounts for deviation from simple exponential drying behavior.

Model performance was assessed from the coefficient of determination (R^2^) and the root mean square error (RMSE) [25,26]. The model giving the highest R^2^ together with the lowest RMSE was selected for each drying temperature, and where these two criteria disagreed, RMSE took precedence [27].

#### 2.2.2. Time Calculation and Sample Preparation for Analysis (Model-Based Sample Preparation)

The model selected in Section 2.2.1 was used to calculate the drying time required to reduce the moisture content of the aged rice to four target stages: 10, 8, 6 and 4% w.b. Samples are identified by the code T*α*–M*β*, where *α* is the drying temperature (°C) and *β* is the target moisture content (% w.b.); for example, T40–M10 denotes aged rice dried at 40 °C to a moisture content of 10% w.b. (Sixteen of the twenty possible temperature–moisture combinations were prepared. The remaining four combinations were omitted because the drying air at those temperatures could not reduce the grain moisture content to the target levels due to equilibrium moisture content limitations.)

After drying for the predicted time, samples were cooled to room temperature in a desiccator, sealed in zip-lock bags and held at 4 °C for 24 h to equilibrate before analysis [22].

### 2.3. Evaluation of Micro-Cracks Using a Light-Transmission Technique

Cracks within the starchy endosperm were examined by light-transmission imaging. Intact kernels from each treatment and from both controls were placed on an LED light panel and illuminated from below [28,29,30]. Kernels were imaged with a stereomicroscope (Stemi 508, ZEISS, Oberkochen, Germany) at 8 magnifications coupled to a digital camera; 20 kernels were examined per condition.

### 2.4. Water Absorption Kinetics and Effective Water Diffusion Coefficient

#### 2.4.1. Peleg’s Hydration Model

Water absorption was measured by soaking 25 g of intact kernels in distilled water at 25 °C [31,32]. At 10, 20, 30, 40, 50 and 60 min, samples were withdrawn, surface water was removed by blotting with absorbent paper, and the samples were weighed immediately before being returned to soak under the same conditions. The data were fitted to the Peleg model:

M_t_ = M_0_ + t/(K_1_ + K_2_t), (7)

where M_t_ is the moisture content at time t (%d.b.), M_0_ is the initial moisture content (%d.b.), K_1_ is Peleg’s rate constant (s/%^1^), related to the initial water absorption rate 1/K_1_, and K_2_ is Peleg’s capacity constant (1/%).

#### 2.4.2. Effective Water Diffusivity (D_eff_)

The effective water diffusivity (D_eff_) during soaking was determined from the analytical solution of Fick’s second law for an infinite cylinder [26], assuming constant kernel dimensions and constant diffusivity throughout soaking:

(8) $$\mathrm{MR}=\frac{4}{\pi^{2}}\sum_{n=1}^{\infty }\frac{1}{\lambda_{n}^{2}}exp\left(-\frac{\lambda_{n}^{2}D_{eff}t}{r^{2}}\right),$$

ln (MR) = ln (0.692) − ((5.783 · D_eff_ · t)/r^2^) (9)

where MR is the dimensionless moisture ratio for water absorption from Equation (1), r is the equivalent radius of the cylindrical rice kernel (m), calculated from the average measured thickness/width of the kernel, t is the soaking time (s), and D_eff_ is the effective water diffusion coefficient (m^2^/s).

D_eff_ is calculated from the slope (Slope, S) of the linear relationship between ln(MR) and time (t), according to Equation (10) [33].

Slope (S) = −5.783D_eff_/r^2^ ⇒ D_eff_ = −S · r^2^/5.783 (10)

### 2.5. Color Measurement and Browning Index

Kernel color was measured with a portable spectrophotometric colorimeter (MiniScan EZ 4500L, HunterLab, Reston, VA, USA), calibrated against black and white standard tiles before each session [28]. Color was recorded in the CIE L*a*b* system [34]. The browning index (BI) was calculated from Equations (11) and (12) [35,36], and the total color difference (ΔE) relative to fresh rice from Equation (13) [14,34]:

BI = 100 × (x − 0.31)/0.17(11)

Where x = (a* + 1.75L*)/(5.645L* + a* − 3.012b*)(12)

ΔE = √((ΔL*)^2^ + (Δa*)^2^ + (Δb*)^2^) (13)

### 2.6. Cooking and Texture Analysis

#### 2.6.1. Cooking

Rice was cooked according to a procedure adapted from ISO 11747:2012 (Rice—Determination of rice kernel resistance to extrusion after cooking). A 20 g sample was combined with 38 g of distilled water (rice-to-water ratio 1:1.9, *w*/*w*) in a 100 mL beaker covered with a watch glass. The beaker was heated in an electric steamer for 20 min and left in the steamer for a further 10 min to allow the heat to distribute evenly. The cooked rice was then transferred to a sealed container and cooled to room temperature for 1 h before testing [37].

#### 2.6.2. Back-Extrusion Test

A 5 g portion taken from the center of each cooked sample was placed in a stainless-steel cylinder and analyzed with a texture analyzer (TA.HD.plus, Stable Micro Systems, Godalming, UK) fitted with a back-extrusion rig. The sample was compressed by a stainless-steel ball probe driven 99 mm into the cylinder, over a total cylinder height of 100 mm, at a probe speed of 1 mm/s. Hardness was recorded as the maximum compression force, and stickiness as the absolute value of the maximum negative force during probe withdrawal.

### 2.7. Multi-Objective Optimization

Thermal pre-treatment conditions were ranked using a weighted linear composite index (CI). This approach is compensatory, so that a favorable value for one attribute can offset an unfavorable value for another [38]. Weights were assigned by the entropy weight method (EWM), which derives weights from the dispersion of the data rather than from expert judgment and therefore removes subjective bias from the weighting step: attributes with greater dispersion carry more information and receive higher weights [39,40].

Four response variables were included: hardness (H), stickiness (S), effective water diffusivity (D_eff_) and total color difference (ΔE). Each variable j was converted to a standardized score z on a 0–1 scale by min–max normalization [41]. Variables to be maximized (S, D_eff_) were normalized by Equation (14) and variables to be minimized (H, ΔE) by Equation (15):

z_ij_ = (x_ij_ − x_j,min_)/(x_j,max_ − x_j,min_) (14)

z_ij_ = (x_j,max_ − x_ij_)/(x_j,max_− x_j,min_) (15)

where x_ij_ is the value of variable j for group i. Hardness and ΔE were minimized to select samples with the greatest reduction in hardness and the smallest color difference from fresh rice; stickiness and D_eff_ were maximized to select samples with the greatest stickiness and the fastest water absorption. Normalization was applied across all eighteen groups (m = 18), comprising the sixteen thermal pre-treatment conditions and the two controls, so that the normalization range and the resulting weights reflect the full quality span between untreated aged rice and the fresh rice against which the treatments were assessed.

The entropy weight of each variable was then calculated from Equations (16)–(18) [39,40]:

(16) $$p_{ij}=z_{ij}/\sum_{i=1}^{m}z_{ij}$$

(17) $$H_{j}=-(1/\ln\;m)\;\sum_{i=1}^{m}p_{ij}\;ln\;p_{ij}$$

(18) $$w_{j}=(1-H_{j})/\;\sum_{j=1}^{n}\left(1-H_{j}\right)$$

where H_j_ is the entropy value of variable j, (1 − H_j_) is its degree of diversification, w_j_ is its weight, m is the number of groups (m = 18) and n is the number of variables (n = 4). In Equation (17), p_ij_ ln p_ij_ was set to zero, where p_ij_ = 0.

The composite index for each group was calculated from Equation (19):

CI_i_ = w_H_ ∙ z_H,i_ + w_S_ ∙ z_S,i_ + w_Deff_ ∙ z_Deff,i_ + w_ΔE_ ∙ z_ΔE,i_ (19)

To express the extent of rejuvenation relative to the starting material, the relative composite index was calculated with untreated aged rice as the baseline (Equation (20)):

relative CI_i_ = CI_i_ − CI_aged rice_ (20)

### 2.8. Statistical Analysis

All measurements were performed in triplicate. Data were analyzed by one-way analysis of variance (ANOVA) followed by Duncan’s multiple range test at *p* < 0.05. Relationships between variables were examined using the Pearson correlation coefficient (*r*). Statistical analyses were performed in IBM SPSS Statistics version 28 (IBM Corp., Armonk, NY, USA). Model fitting, correlation analysis, weight calculation, composite index computation and graph plotting were carried out in MATLAB R2023a (MathWorks, Natick, MA, USA).

## 3. Results and Discussion

### 3.1. Drying Kinetics and Mathematical Modeling Parameters

Moisture content decreased non-linearly with drying time at all five temperatures (Figure 1), following the pattern typical of biological materials [33,42]. The rate of moisture removal increased with hot-air temperature, and raising the temperature from 40 to 120 °C shortened the time required to reach each target moisture content point. This behavior reflects the greater thermal energy transferred to the kernel at higher temperatures, which raises the kinetic energy of the water molecules and weakens the binding forces between water and the internal kernel structure. The effective water diffusivity increases accordingly, so that water migrates from the kernel core to the surface and evaporates more rapidly [33,43].

Among the five models tested, the Two-term and Page models described the drying curves most accurately across the full temperature range (R^2^ > 0.982, RMSE < 0.039). The fitted equations were therefore used to predict the drying time required to reach target moisture contents of 10, 8, 6 and 4% w.b., and these predicted times determined the drying endpoint for the samples used in all subsequent analyses.

### 3.2. Crack Morphology and Kernel Appearance

Light-transmission imaging (Figure 2) showed that both controls, fresh rice and untreated aged rice, had a translucent, smooth endosperm with no visible surface cracks. All thermally pre-treated samples developed fine cracks on the kernel surface and in the subsurface layer. These formed an interconnected network resembling a desiccation-crack pattern distributed across the kernel surface, rather than large fractures traversing the kernel core. Based on the visual percentage scale proposed by Mee-ngern et al. [28], these were semi-quantitatively graded as 100% surface cracking. However, it should be noted that detailed digital image metrics (such as crack density or area fraction) were not quantified in this study, and these visual observations should be interpreted cautiously when relating crack patterns to functional quality attributes. The pattern was similar across the drying temperatures (40–120 °C) and target moisture contents (4–10% w.b.) examined. In the samples dried at 100 and 120 °C to 4% w.b., however, the cracks were less clearly resolved: heat-induced opacification of the kernel, similar in appearance to chalkiness, reduced the visual contrast and partially obscured the surface cracks.

Cracking of this kind arises from internal tensile stress generated by moisture and temperature gradients during heating [28,43]. Water evaporates from the kernel surface considerably faster than it diffuses from the interior to the surface [44,45]. The resulting moisture difference between layers causes the surface to contract, and tensile stress accumulates within the kernel [43,46]. At 40–60 °C, moisture is lost more slowly than at higher temperatures, but the continuous reduction in moisture content still generates sufficient surface stress to produce the same cracking pattern [28]. The opacification observed at the highest temperatures is consistent with the increase in whiteness and loss of transparency previously reported after thermal pre-treatment of aged rice [21].

### 3.3. Water Soaking Kinetics and Diffusivity

Sequential imaging of water penetration during soaking (Figure 3), in which the degree of opacity indicates the extent of water uptake, showed that untreated kernels required approximately 40 min for water to pass through the surface layer and reach the kernel interior. In thermally pre-treated kernels, water reached the kernel core within 5–10 min. The cracks formed during pre-treatment opened pathways through which water rapidly penetrated the endosperm [31,47].

The Peleg parameters are given in Table 1. Values of 1/K_1_ in the thermally pre-treated samples ranged from 0.099 ± 0.006 %/s (T120–M8) to 0.198 ± 0.025 %/s (T80–M4) and were significantly higher than those of fresh rice (0.041 ± 0.006 %/s) and untreated aged rice (0.026 ± 0.002 %/s) (*p* < 0.05). The highest values occurred consistently at the lowest target moisture content. Because 1/K_1_ represents the initial water absorption rate, these values indicate that pre-treatment accelerated the early stage of hydration [48]. Two factors likely contribute to this effect. The surface cracks function as preferential pathways that reduce diffusion resistance at the kernel surface, allowing water to enter the endosperm more readily. In addition, the lower initial moisture content of the pre-treated kernels creates a larger moisture gradient between the kernel and the soaking water, which increases the driving force for water uptake during the initial soaking period [49].

D_eff_ in the thermally pre-treated samples ranged from 1.04 ± 0.00 to 1.35 ± 0.00 × 10^−10^ m^2^/s and was significantly higher than in fresh rice (0.76 ± 0.00 × 10^−10^ m^2^/s) and untreated aged rice (0.63 ± 0.11 × 10^−10^ m^2^/s) (*p* < 0.05), consistent with the expected increase in water diffusion when voids or a porous structure are introduced into the kernel. Untreated aged rice had the lowest D_eff_ of all samples. This may be attributed to the aging process, during which the protein network surrounding the starch granules becomes more tightly bound through disulfide bonds and ferulic acid cross-links forming at the cell wall, increasing rigidity and restricting the natural pathways for water diffusion [31,50,51].

### 3.4. Thermal Discoloration and Color Attributes (ΔE, $L^{*}$, $a^{*}$, $b^{*}$, BI)

Thermal pre-treatment significantly affected kernel color (*p* < 0.05), as shown by the CIE L*a*b* coordinates and browning index (BI) in Table 2 and the total color difference (ΔE) in Figure 4.

L* increased in all pre-treated samples, from 57.28 ± 0.57 in untreated aged rice to between 62.72 ± 0.44 (T40–M10) and 68.59 ± 0.43 (T100–M4). This increase is attributed primarily to changes in light reflection and scattering at the kernel surface caused by the crack network and by the reduced moisture content [21,34]. At the higher drying temperatures, a second mechanism is likely to contribute: KDML 105 has a gelatinization onset temperature of approximately 70–78 °C [52], and the endosperm of milled rice consists predominantly of starch granules, which account for 80–90% of the dry weight [53]. Pre-treatment at elevated temperatures may induce localized, surface-level starch alterations, as described in previous studies where thermal treatment near the gelatinization range altered optical opacity and whiteness [34,54]. However, because internal thermal phase transitions and crystalline modifications were not directly quantified in this study, the observed rise in L* is attributed primarily to physical changes in surface light scattering from dehydration and crack formation, whereas partial gelatinization remains a literature-supported hypothesis for the high-temperature treatments.

The redness–greenness value (a*) of the pre-treated samples was negative throughout, ranging from −0.24 ± 0.02 to −0.40 ± 0.06, and thus closer to zero than in untreated aged rice (−0.59 ± 0.03) and fresh rice (−0.52 ± 0.05). Yellowness (b*) ranged from 11.51 ± 0.31 to 13.16 ± 0.23 and was significantly higher than in untreated aged rice (11.33 ± 0.50) and fresh rice (10.01 ± 0.36) (*p* < 0.05), with the highest values at the lowest target moisture content. Despite the rise in b*, BI in the pre-treated samples (19.23 ± 0.56 to 21.08 ± 0.57) remained close to that of untreated aged rice (20.74 ± 1.29), indicating that the drying conditions applied did not induce browning beyond the level already present in the aged rice. *BI* did nonetheless exceed that of fresh rice (17.90 ± 0.84) in every pre-treated sample.

Relative to fresh rice, ΔE ranged from 5.37 ± 0.35 (T40–M10) to 11.14 ± 0.44 (T100–M4). All values exceeded 3.0, the threshold above which a color difference is readily perceptible to the human eye [34]. The largest differences occurred at low target moisture contents (4 and 6% w.b.) combined with high drying temperatures (100 and 120 °C), reflecting the combined effect of temperature on reaction rates and of the steep moisture gradient [33,42]. T40–M10 was the condition closest in color to fresh rice. The overall increase in ΔE was driven principally by the rise in L* [34]. Because this shift reflects increased lightness rather than browning, its commercial significance is likely to be limited: in consumer testing of KDML 105 and its mutants, color and appearance liking formed a secondary preference dimension relative to aroma, taste and texture, and a sample that differed significantly from KDML 105 in color liking was still rated equally for overall liking [55]. Direct consumer testing of the pre-treated rice would nevertheless be required to confirm commercial acceptability.

### 3.5. Textural Properties and Cooking Quality (Hardness and Stickiness)

Thermal pre-treatment significantly altered the texture of the cooked rice (*p* < 0.05, Figure 5 and Figure 6). Hardness across all samples spanned 16.28–25.11 N, bounded by the two controls; untreated aged rice (25.09 ± 1.71 N) reflected the firm texture that develops during aging, whereas fresh rice (16.70 ± 0.68 N) lay at the opposite end of the range.

Among the pre-treated samples, target moisture content was the dominant factor governing hardness [54]. Drying to 10% w.b. reduced hardness to 16.28–18.53 N at every drying temperature. The lowest value was obtained at T40–M10 (16.28 ± 1.01 N), followed by T100–M10 (16.47 ± 1.09 N) and T60–M10 (16.96 ± 1.01 N); none of these differed significantly from fresh rice (*p* > 0.05). Drying to 4% w.b. raised hardness to 23.27–25.11 N, with the highest value at T80–M4 (25.11 ± 1.62 N). Samples in this group did not differ significantly from untreated aged rice, indicating that the reduction in hardness achieved at 10% w.b. was lost entirely when drying continued to 4% w.b.

The reduction in hardness at 10% w.b. is consistent with the formation of surface cracks that accelerate water penetration to the kernel core during cooking, allowing the starch granules to hydrate and swell more completely and yielding a softer cooked texture [25,47]. The return of hardness at 4% w.b. may be attributed to changes in the starch granule structure induced by the more severe thermal load required to reach that moisture content [56].

Stickiness followed a different pattern. Fresh rice showed the highest stickiness (12.73 ± 0.71 N) and untreated aged rice the lowest (5.06 ± 0.65 N). Values for the pre-treated samples ranged from 5.79 to 7.70 N, a slight increase relative to untreated aged rice but with no significant differences among drying temperatures or target moisture contents, and all remained significantly below that of fresh rice. Although the present study was confined to instrumental texture analysis—specifically, the stickiness of cooked kernels measured as peak adhesion force (N)—the observed pattern can be rationalized in terms of starch-solubilization mechanisms that are well documented in the literature. Stickiness in cooked rice is attributed to soluble starch, chiefly amylopectin and short-chain amylose, which leaches from the kernel during cooking and coats its surface [47,57]. The present pre-treatment accelerated water absorption and restored hardness but, based on the evidence available here, did not raise stickiness to the level of fresh rice; this is consistent with the interpretation that the treatment did not enhance the solubility or release of these surface-forming molecules to a degree comparable with fresh rice [47,57]. A further contributing factor, as reported elsewhere, is that aging promotes the formation of amylose-lipid complexes, which restrict starch swelling and solubilization [13,18]; a physical pre-treatment that fractures the kernel surface would not be expected to dissociate these complexes. As no starch composition or leaching measurements were undertaken in this work, these mechanisms are offered as literature-based context for the texture data rather than as direct experimental findings.

### 3.6. Pearson Correlation Analysis and Physical Mechanisms

Pearson correlation coefficients between the quality attributes of cooked rice and the kinetic parameters are shown in Figure 7. Hardness correlated positively with 1/K_1_ (*r* = 0.837), ΔE (*r* = 0.879), L* (*r* = 0.867) and b* (*r* = 0.805). The positive association between hardness and 1/K_1_ appears to contradict the expected relationship between water absorption and softening, but both variables increase as the target moisture content decreases and are therefore confounded by drying severity.

Drying to a very low moisture content produces a large moisture gradient between the kernel and the soaking water, creating a strong driving force for initial water uptake and raising 1/K_1_ [47,58]. Concurrently, the same severe drying conditions impose a higher thermal load that alters the starch-protein biopolymers [56]. Partial cooking of surface/subsurface starch granules (similar to parboiling) creates a denser, more crystalline kernel structure [34,54]. Consequently, this heat-induced structural reinforcement overrides the potential softening effect of rapid initial water uptake, causing the rice to remain hard despite fast initial hydration kinetics.

D_eff_ correlated weakly with hardness (*r* = 0.373) and strongly with 1/K_1_ (*r* = 0.706), suggesting that D_eff_ reflects sustained water transport into the endosperm through the crack network rather than initial surface uptake alone [31,47]. Cracking induced at 10% w.b. was therefore associated with a higher D_eff_ and a reduction in hardness to the level of fresh rice [19,30]. Stickiness correlated weakly with hardness (*r* = 0.305) and weakly with all color and kinetic parameters (*r* = −0.185 to 0.306), consistent with stickiness being governed by the release of soluble starch during cooking—a process largely independent of the physical water-diffusion and cracking mechanisms that determine hardness [59].

### 3.7. Multi-Objective Optimization for Aged Rice Rejuvenation

The entropy weights calculated for the four response variables are given in Table 3.

Composite index scores for the sixteen pre-treatment conditions and the two controls are shown in Figure 8. Fresh rice obtained the highest score (CI = 0.913). Among the pre-treated samples, T40–M10 ranked highest (CI = 0.509), followed by T100–M10 (0.502), T80–M10 (0.490) and T60–M10 (0.456); T100–M4 ranked lowest (0.103). The ranking indicates that a target moisture content of 10% w.b. produced the best balance among the four response variables at every drying temperature. T40–M10 combined the lowest hardness of any sample, the smallest color difference (ΔE = 5.37) and a high D_eff_ (1.32 × 10^−10^ m^2^/s), and therefore scored highest on the two most heavily weighted criteria other than stickiness. Drying to 4% w.b. accelerated initial water uptake but increased hardness sharply, which lowered the scores of that group. Because no condition increased stickiness appreciably, all pre-treated samples scored well below fresh rice.

However, an inherent limitation of the entropy-weighting approach is that criteria weights are determined strictly by mathematical data dispersion rather than consumer sensory preferences. Because stickiness could not be restored under any of the tested thermal treatments, its relative variance across pre-treated samples remained small, causing the index to rank treatments based primarily on physical and kinetic improvements. From a sensory perspective, the failure to recover stickiness represents a major barrier, as stickiness is a primary driver of consumer acceptability in cooked rice.

To express the extent of rejuvenation, CI scores were recalculated relative to untreated aged rice as a baseline (relative CI, Figure 9). Fresh rice gave a relative CI of +0.522, defining the full quality gap between aged and fresh rice. Six conditions moved toward fresh rice: T40–M10 (+0.118), T100–M10 (+0.111), T80–M10 (+0.098), T60–M10 (+0.065), T100–M8 (+0.027) and T120–M10 (+0.005). Five of these had a target moisture content of 10% w.b. The remaining conditions gave negative relative CI values, indicating that they shifted the properties of the rice further from those of fresh rice, an effect resembling accelerated aging rather than rejuvenation [20,60]. Even the highest-ranked condition recovered less than a quarter of the gap between aged and fresh rice.

These results indicate that the thermal rejuvenation of aged rice depends primarily on limiting the thermal load, with a target moisture content of 10% w.b. giving the most consistent improvement across drying temperatures. Below this level, the increase in hardness outweighed the benefit of faster water absorption, which may reflect changes in the starch structure induced by the greater thermal load. The extent of rejuvenation achievable by this approach was nonetheless bounded by the stickiness response, which did not improve under any condition tested.

From an industrial scalability perspective, the optimal T40–M10 treatment (40 °C drying to 10% w.b.) offers low energy intensity and minimal risk of severe thermal cracking compared to higher-temperature treatments. The process can be readily implemented using conventional commercial grain dryers (e.g., recirculating batch or continuous cross-flow dryers). However, the primary operational constraint is processing time; drying at 40 °C down to 10% w.b. requires longer residence times, reducing continuous throughput unless balanced by optimized air velocity or two-stage drying protocols.

## 4. Conclusions

Thermal pre-treatment of aged KDML 105 rice by hot-air drying induced a network of surface cracks under all sixteen conditions examined. These cracks increased the effective water diffusivity and shortened the time required for water to reach the kernel core during soaking from approximately 40 min to 5–10 min.

The endpoint moisture content, rather than the drying temperature, determined the textural outcome. Drying to 10% w.b. reduced the hardness of the cooked rice to a level not significantly different from fresh rice at every temperature tested, whereas drying to 4% w.b. returned hardness to the level of untreated aged rice. Stickiness increased only slightly and remained significantly below that of fresh rice under all conditions. Thermal pre-treatment increased kernel lightness and yellowness without raising the browning index above the level already present in aged rice, but the total color difference relative to fresh rice exceeded the threshold of visual perception in every case, and was smallest under the mildest condition.

Multi-objective optimization using a weighted linear composite index identified T40–M10 as the highest-ranked condition (CI = 0.509, relative CI = +0.118). Six conditions shifted the properties of aged rice toward those of fresh rice, five of them at a target moisture content of 10% w.b. The remaining conditions shifted the properties further away, resembling accelerated aging rather than rejuvenation.

This study evaluated rejuvenation through physical and textural attributes—hardness, stickiness, color, and water-absorption and drying kinetics—without assessing accompanying changes in nutritional composition or functional properties; these represent an important direction for future work, given the established links between starch fine structure and eating quality noted in the Introduction. Rejuvenation was likewise assessed instrumentally, without evaluation of aroma retention or consumer sensory acceptance; the treatment is therefore not proposed as a means of restoring the characteristic aroma of KDML 105.

Aged rice can therefore be partially rejuvenated by thermal pre-treatment, provided drying is terminated at a moisture content of about 10% w.b. Recovery was limited to hardness; stickiness did not improve under any condition tested, and restoring it is likely to require a treatment acting on the solubilization of starch rather than on the physical structure of the kernel alone.

## Figures and Tables

**Figure 1:**
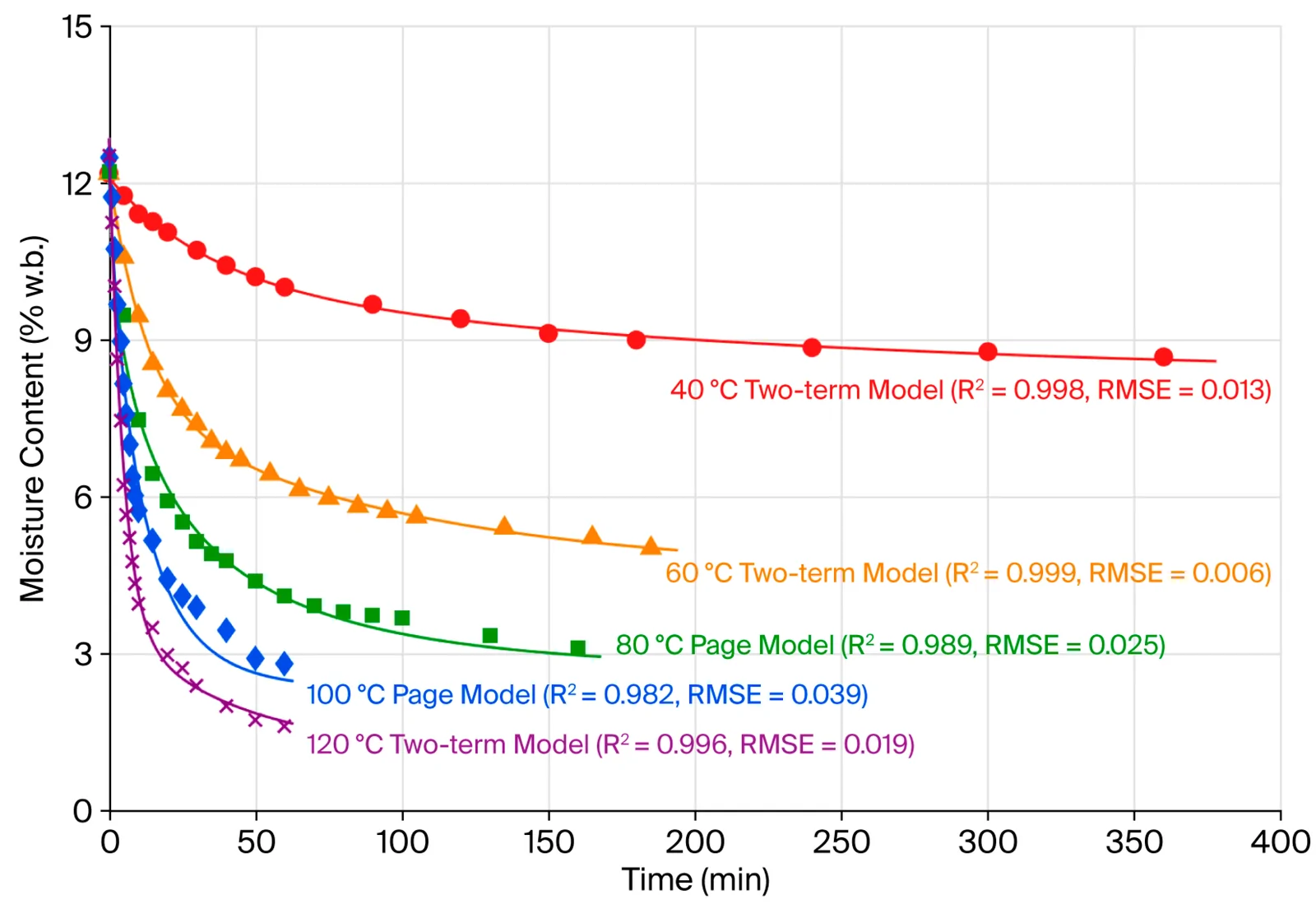
Drying curves and fitted models for aged rice at 40–120 °C.

**Figure 2:**
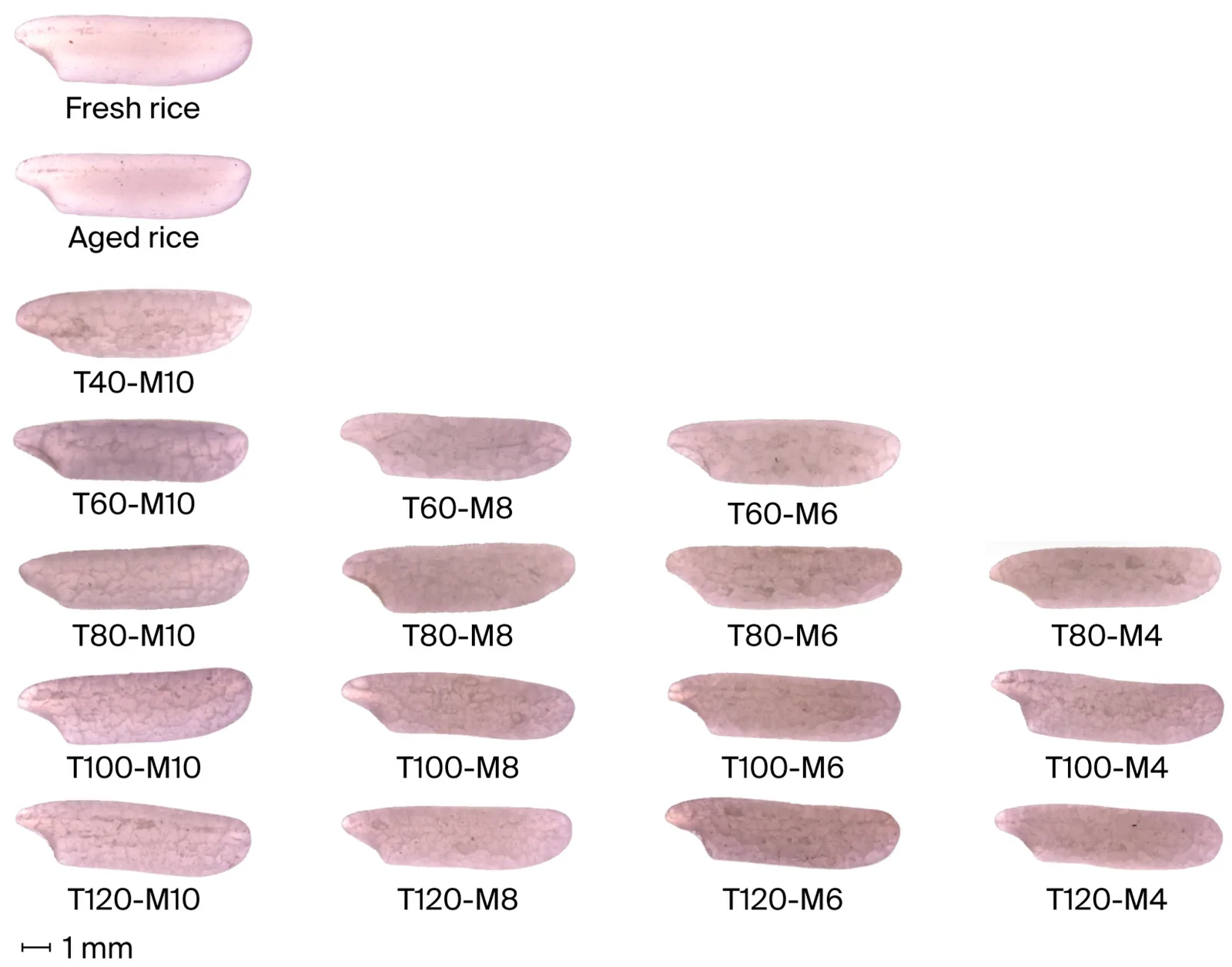
Light-transmission images of KDML 105 kernels: fresh rice (0% visual surface crack grade), untreated aged rice (0% visual surface crack grade), and aged rice after thermal pre-treatment (100% visual surface crack grade) [28].

**Figure 3:**
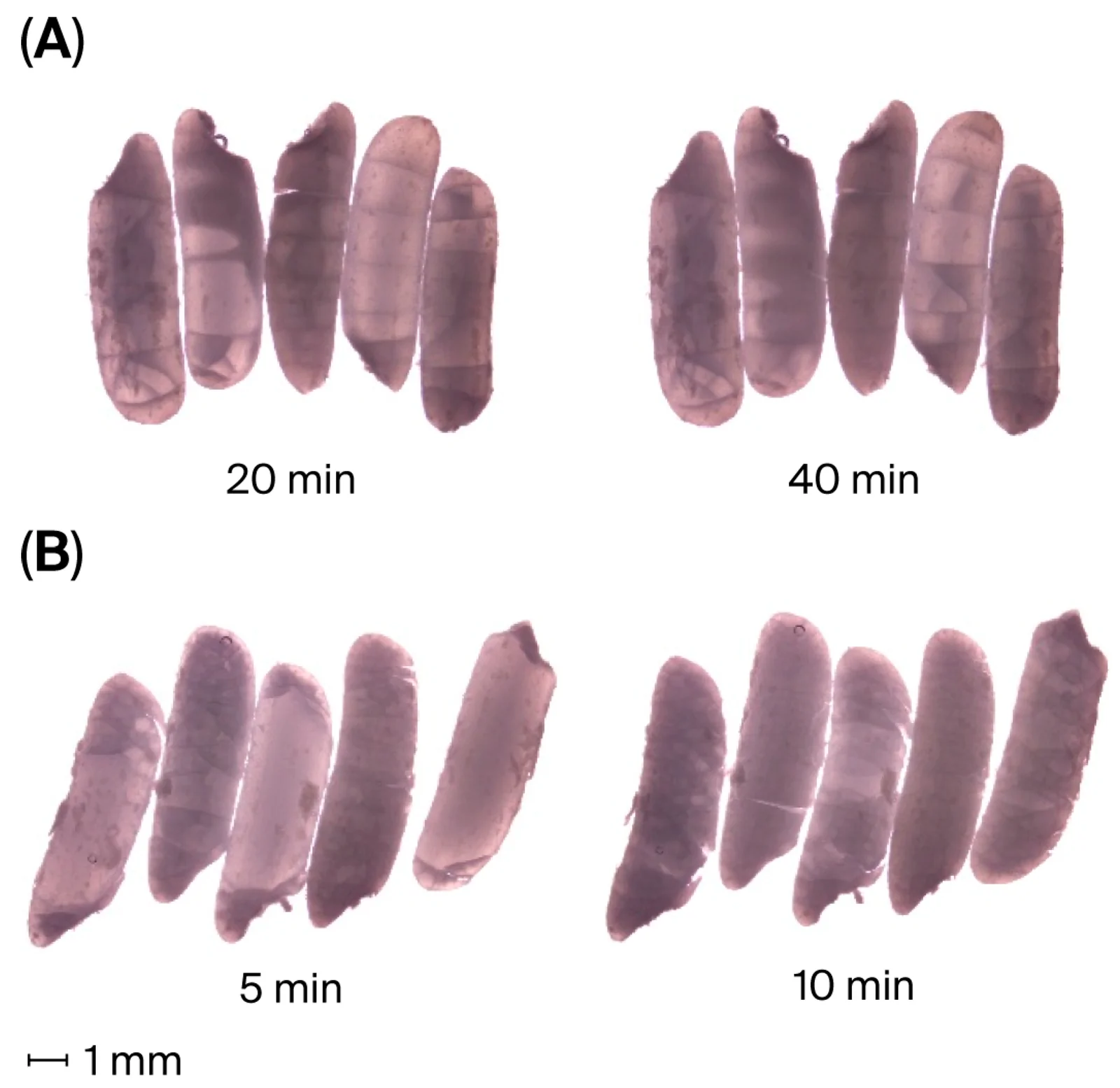
Water penetration during soaking in (**A**) untreated and (**B**) thermally pre-treated kernels.

**Figure 4:**
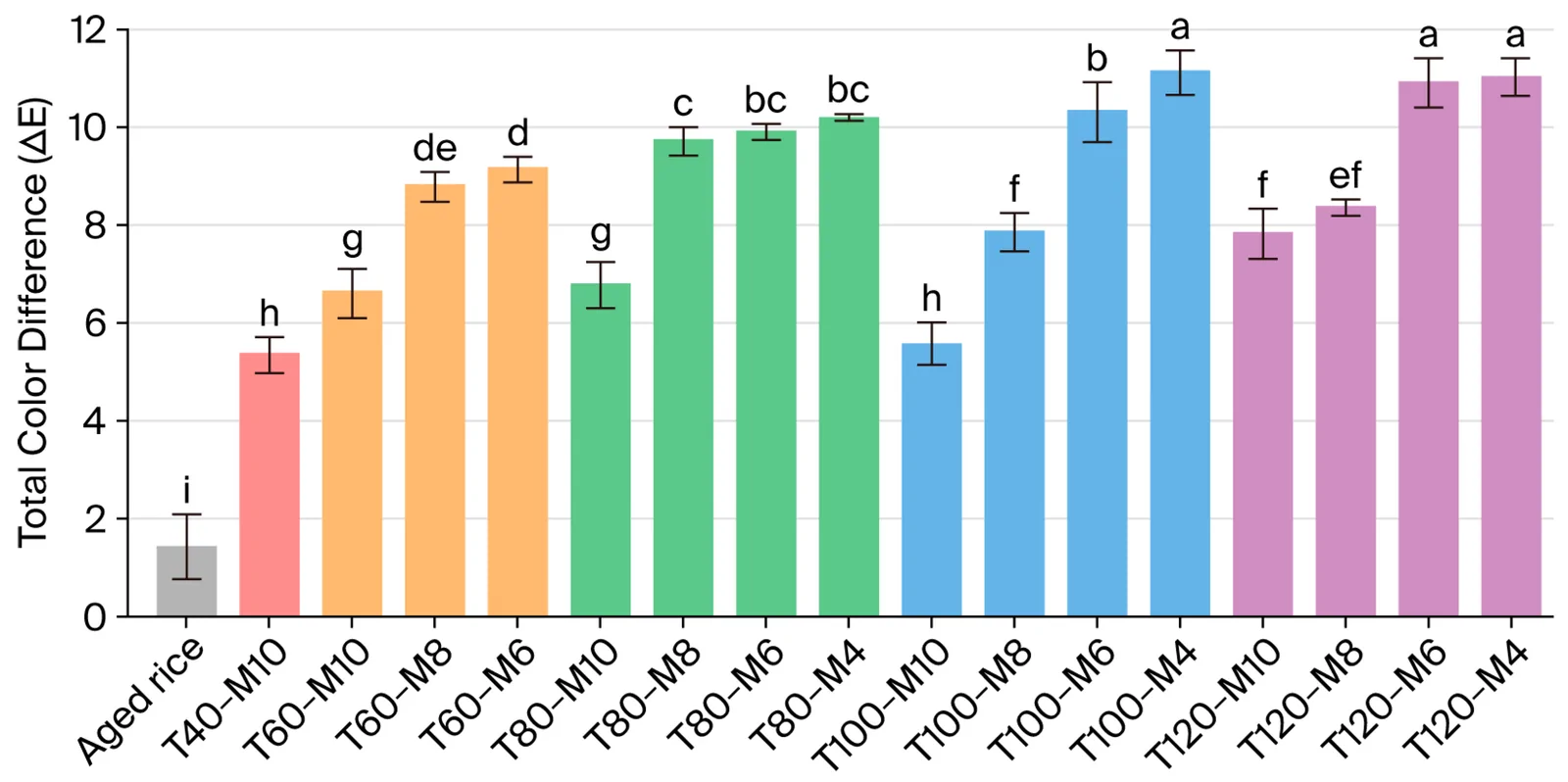
Total color difference (ΔE) relative to fresh rice. Error bars represent standard deviation of the data (n = 3). Different letters indicate significant differences (*p* < 0.05).

**Figure 5:**
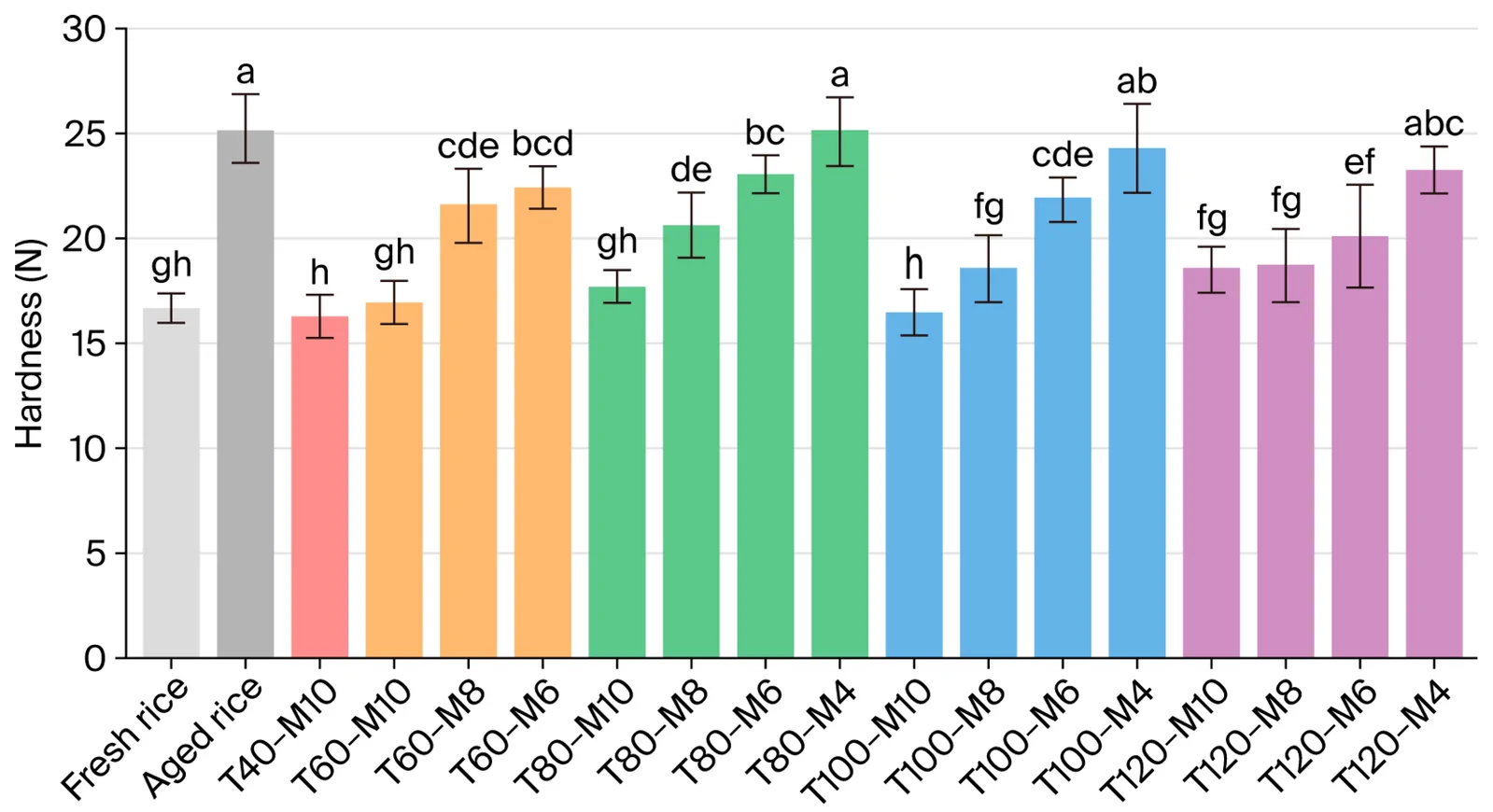
Hardness of cooked rice. Error bars represent standard deviation of the data (n = 3). Different letters indicate significant differences (*p* < 0.05).

**Figure 6:**
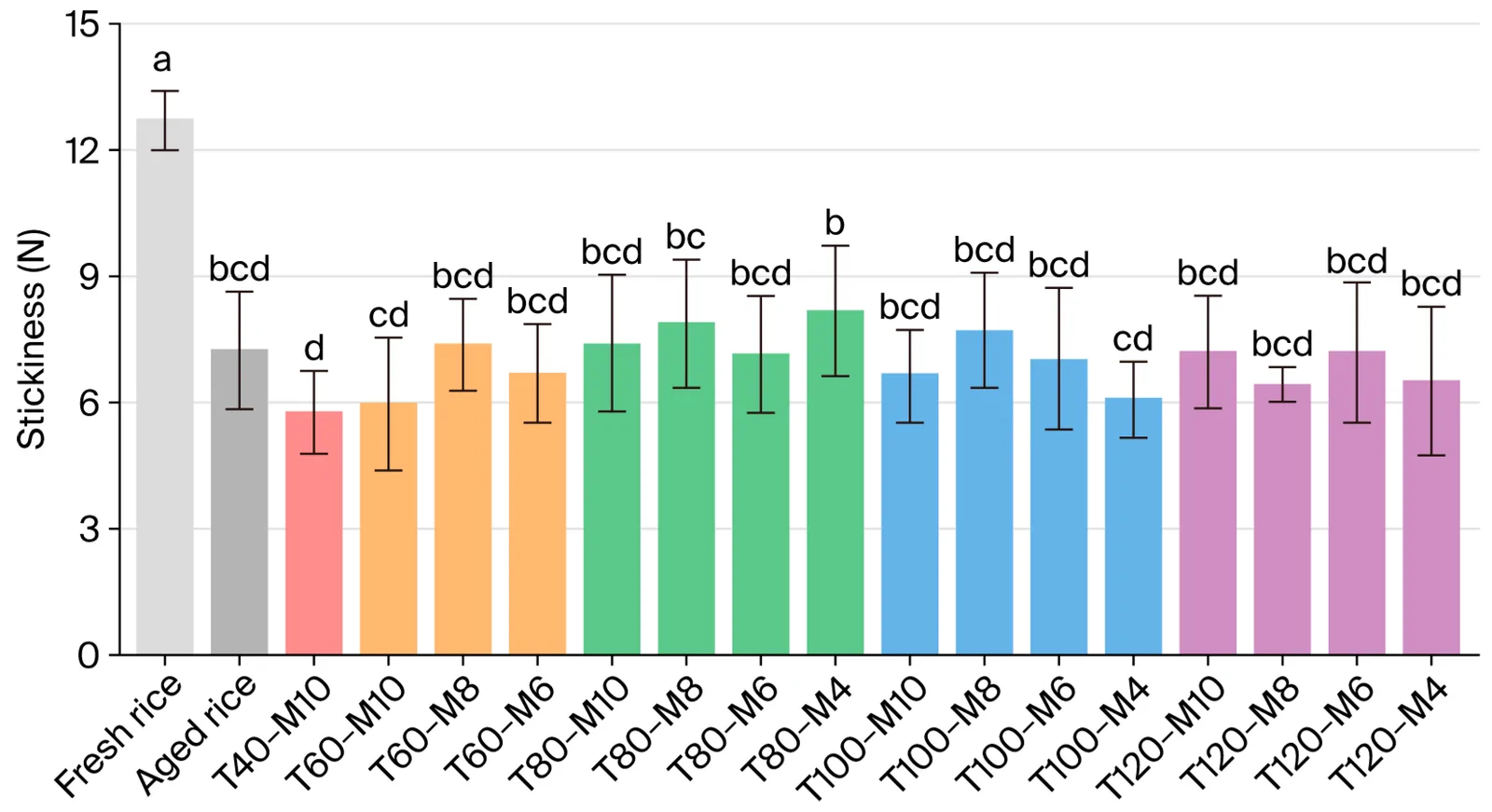
Stickiness of cooked rice. Error bars represent standard deviation of the data (n = 3). Different letters indicate significant differences (*p* < 0.05).

**Figure 7:**
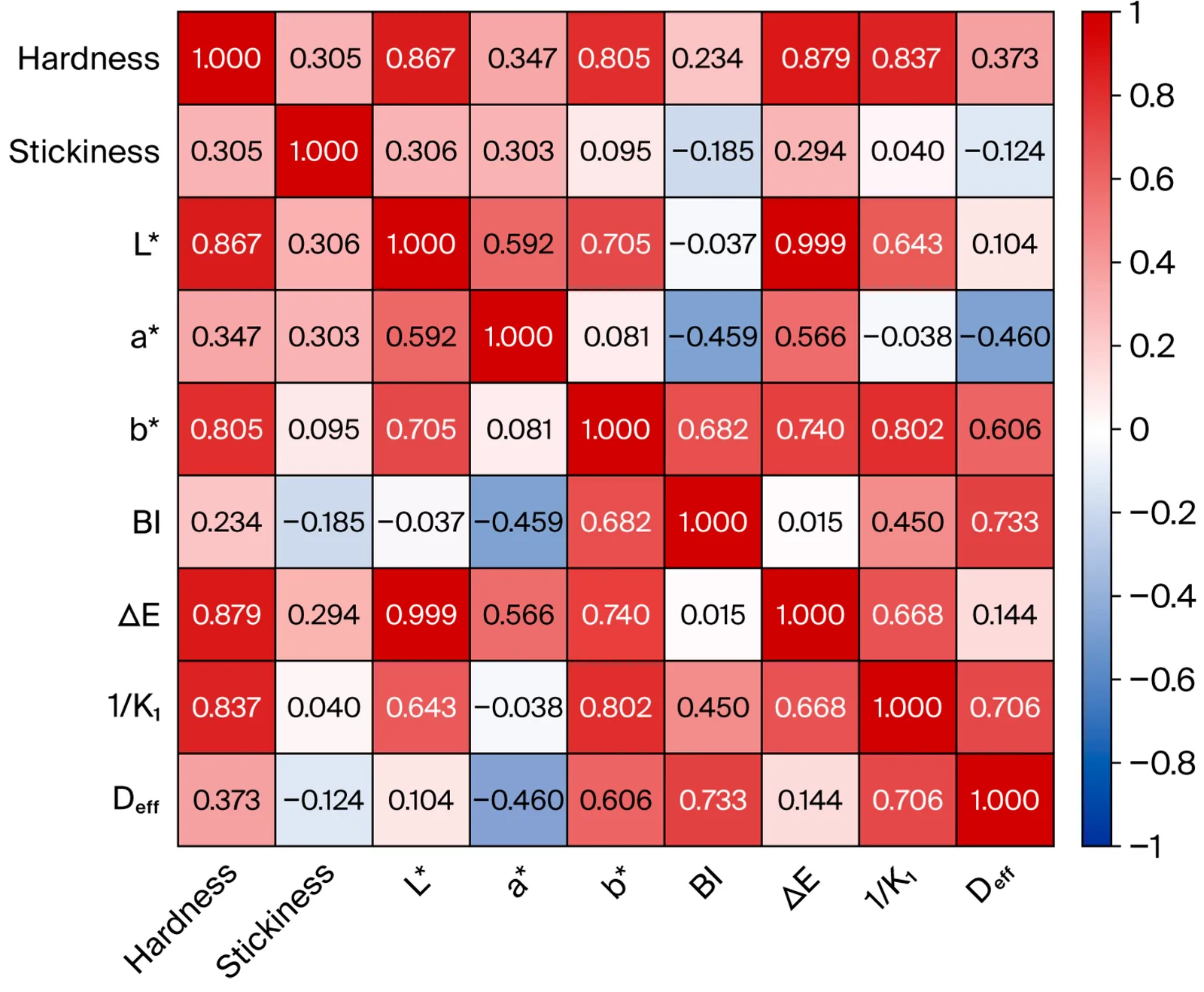
Pearson correlation matrix of quality and kinetic attributes.

**Figure 8:**
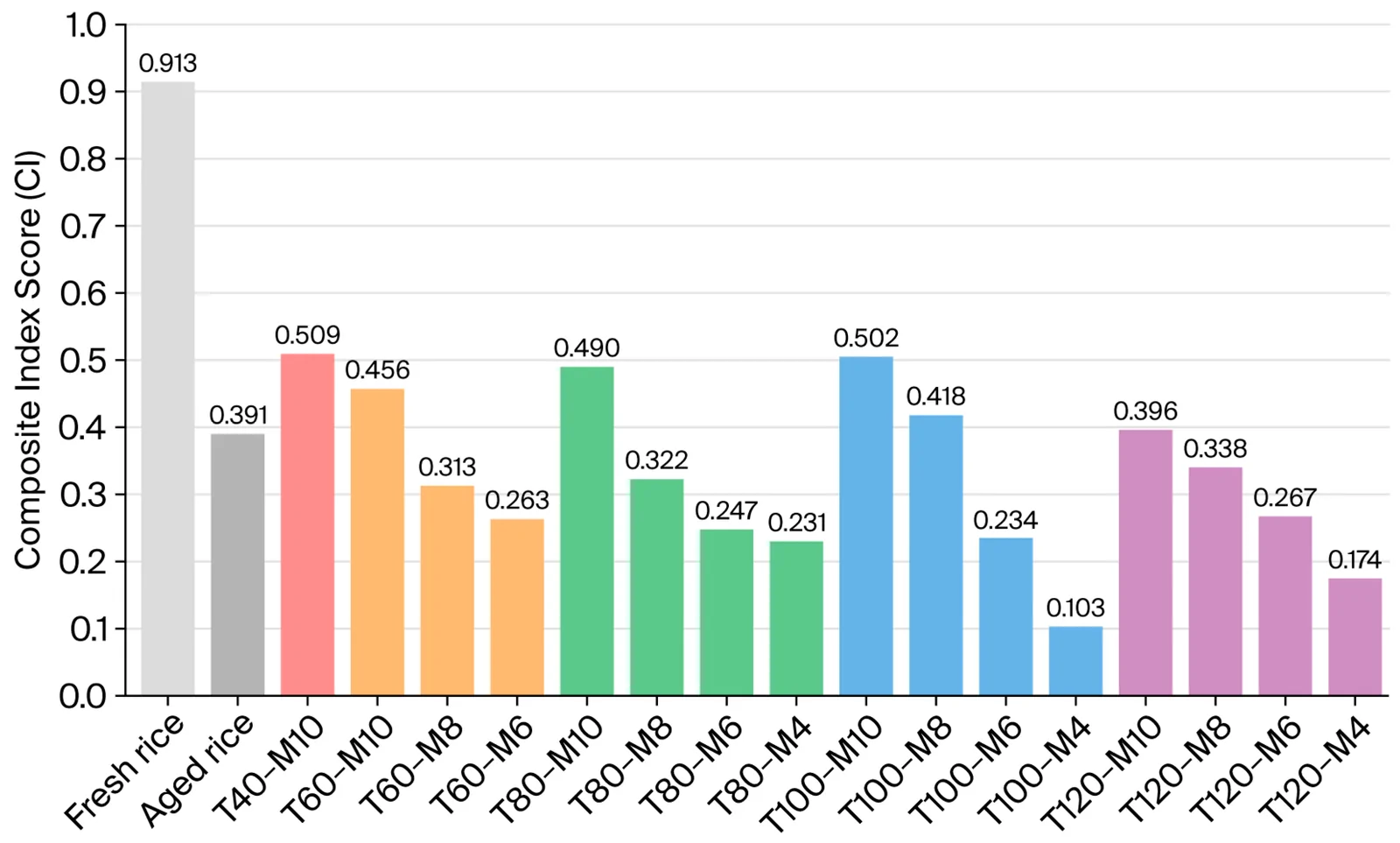
Composite index (CI) scores of all treatments and controls.

**Figure 9:**
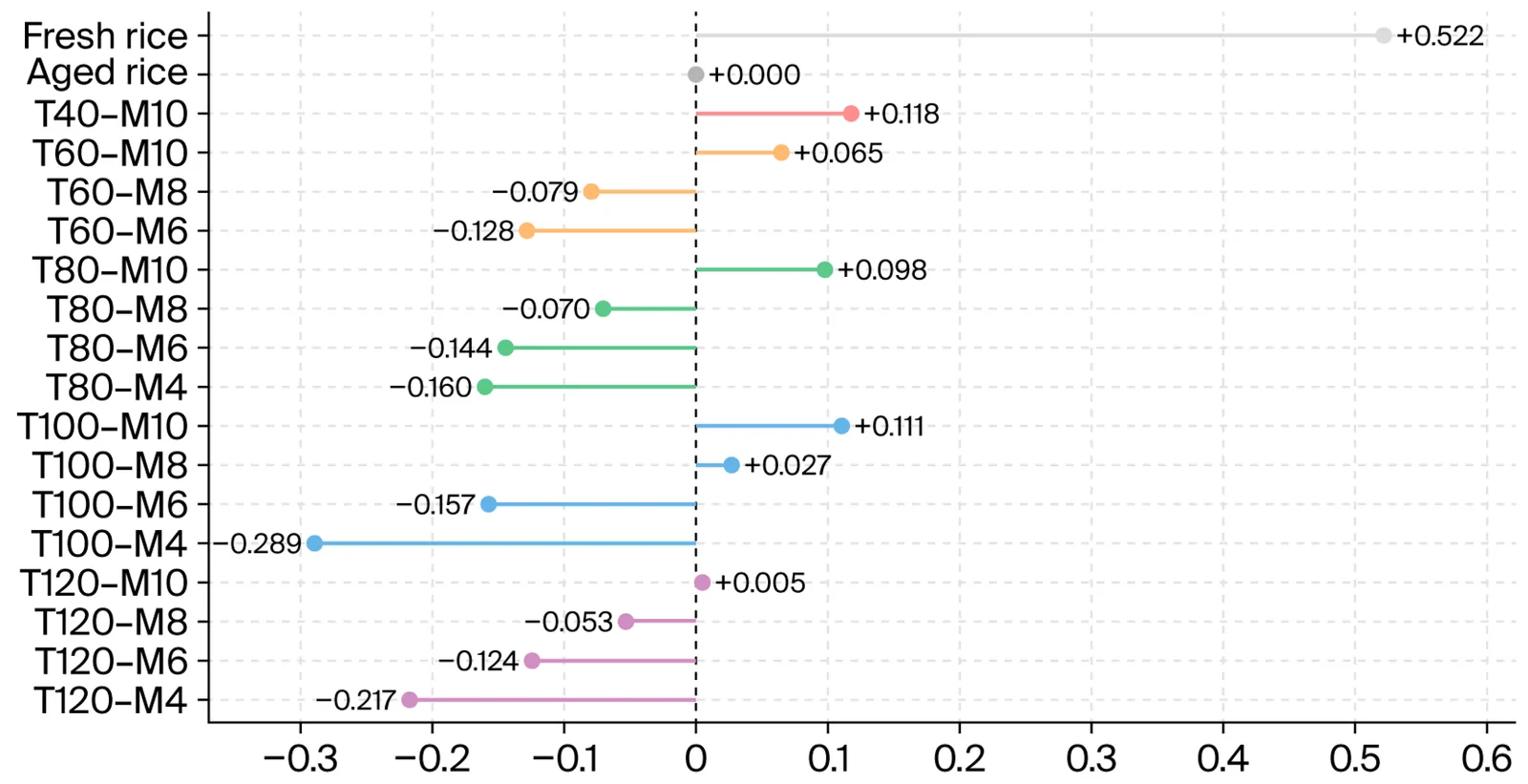
Relative composite index scores with reference to untreated aged rice.

**Table 1:** Water absorption parameters of fresh, aged and thermally pre-treated KDML 105 rice.

Treatment	Diffusion Properties	
	1/K_1_ (%/s)	D_eff_ (×10^−10^ m^2^/s)
Control		
Fresh rice	0.041 ± 0.006 f	0.76 ± 0.00 f
Aged rice	0.026 ± 0.002 f	0.63 ± 0.11 g
Heat Treatment		
T40–M10	0.133 ± 0.001 bc	1.32 ± 0.00 ab
T60–M10	0.128 ± 0.005 bcd	1.30 ± 0.12 ab
T60–M8	0.134 ± 0.004 bc	1.19 ± 0.11 bcd
T60–M6	0.148 ± 0.006 b	1.30 ± 0.12 ab
T80–M10	0.128 ± 0.010 bcd	1.26 ± 0.11 abc
T80–M8	0.116 ± 0.004 cde	1.14 ± 0.00 cde
T80–M6	0.176 ± 0.015 a	1.35 ± 0.00 a
T80–M4	0.198 ± 0.025 a	1.35 ± 0.00 a
T100–M10	0.100 ± 0.003 e	1.06 ± 0.00 de
T100–M8	0.110 ± 0.012 cde	1.06 ± 0.00 de
T100–M6	0.148 ± 0.020 b	1.15 ± 0.10 cde
T100–M4	0.185 ± 0.026 a	1.16 ± 0.10 cde
T120–M10	0.106 ± 0.004 de	1.04 ± 0.00 e
T120–M8	0.099 ± 0.006 e	1.04 ± 0.00 e
T120–M6	0.144 ± 0.027 b	1.16 ± 0.10 cde
T120–M4	0.177 ± 0.023 a	1.34 ± 0.00 a

Values in the table represent means ± standard deviation. Different letters within the same column indicate a statistically significant difference (*p* < 0.05).

**Table 2:** Color attributes of fresh, aged and thermally pre-treated KDML 105 rice.

Treatment	Color Attributes			
	L*	a*	b*	BI
Control				
Fresh rice	57.82 ± 0.38 i	−0.52 ± 0.05 f	10.01 ± 0.36 i	17.90 ± 0.84 e
Aged rice	57.28 ± 0.57 i	−0.59 ± 0.03 g	11.33 ± 0.50 h	20.74 ± 1.29 ab
Heat Treatment				
T40–M10	62.72 ± 0.44 g	−0.40 ± 0.06 e	12.16 ± 0.37 def	20.57 ± 0.87 abc
T60–M10	63.93 ± 0.57 f	−0.36 ± 0.04 de	12.57 ± 0.23 bcd	20.96 ± 0.57 a
T60–M8	66.24 ± 0.31 d	−0.33 ± 0.05 bcd	12.54 ± 0.29 bcd	20.12 ± 0.55 abcd
T60–M6	64.43 ± 0.76 f	−0.29 ± 0.04 abcd	13.08 ± 0.29 a	21.08 ± 0.57 a
T80–M10	64.33 ± 0.53 f	−0.31 ± 0.06 abcd	11.92 ± 0.39 fg	19.66 ± 0.83 cd
T80–M8	67.25 ± 0.34 c	−0.26 ± 0.11 ab	12.42 ± 0.27 bcdef	19.65 ± 0.66 cd
T80–M6	67.39 ± 0.27 c	−0.27 ± 0.07 abc	12.58 ± 0.43 bcd	19.89 ± 0.91 bcd
T80–M4	67.52 ± 0.04 c	−0.35 ± 0.02 cde	13.16 ± 0.23 a	20.77 ± 0.44 ab
T100–M10	63.20 ± 0.54 g	−0.32 ± 0.06 bcd	11.51 ± 0.31 gh	19.25 ± 0.83 d
T100–M8	65.37 ± 0.41 e	−0.31 ± 0.05 abcd	12.18 ± 0.29 def	19.79 ± 0.55 bcd
T100–M6	67.85 ± 0.57 bc	−0.26 ± 0.05 ab	12.46 ± 0.32 bcde	19.51 ± 0.37 cd
T100–M4	68.59 ± 0.43 a	−0.28 ± 0.06 abc	12.80 ± 0.28 ab	19.86 ± 0.50 bcd
T120–M10	65.31 ± 0.44 e	−0.24 ± 0.02 a	12.27 ± 0.32 cdef	20.03 ± 0.49 abcd
T120–M8	65.95 ± 0.11 d	−0.29 ± 0.04 abcd	11.97 ± 0.32 ef	19.23 ± 0.56 d
T120–M6	68.40 ± 0.45 ab	−0.28 ± 0.05 abcd	12.72 ± 0.44 abc	19.79 ± 0.69 bcd
T120–M4	68.39 ± 0.35 ab	−0.30 ± 0.04 abcd	13.15 ± 0.52 a	20.53 ± 0.92 abc

Values in the table represent means ± standard deviation. Different letters within the same column indicate a statistically significant difference (*p* < 0.05).

**Table 3:** Entropy weights of the four response variables.

Factor	Hardness	Stickiness	ΔE	D_eff_
H_j_	0.913	0.881	0.855	0.964
1 − H_j_	0.087	0.118	0.145	0.036
w_i_	0.226	0.306	0.375	0.092

## Data Availability

The original contributions presented in this study are included in the article. Further inquiries can be directed to the corresponding author.

## Abbreviations

The following abbreviations are used in this manuscript:

KDML 105	Khao Dawk Mali 105 rice
AOAC	Association of Official Analytical Collaboration International
ISO	International Organization for Standardization
